# A Novel Event-Based Incipient Slip Detection Using Dynamic Active-Pixel Vision Sensor (DAVIS)

**DOI:** 10.3390/s18020333

**Published:** 2018-01-24

**Authors:** Amin Rigi, Fariborz Baghaei Naeini, Dimitrios Makris, Yahya Zweiri

**Affiliations:** 1 Faculty of Science, Engineering and Computing, Kingston University London, London SW15 3DW, UK; gamin009@gmail.com (A.R.); k1547381@kingston.ac.uk (F.B.N.); d.makris@kingston.ac.uk (D.M.); 2 Robotics Institute, Khalifa University of Science and Technology, P.O. Box 127788, Abu Dhabi 999041, UAE

**Keywords:** incipient slip, robot grasping, dynamic vision sensor, tactile sensor, vision-based slip detection

## Abstract

In this paper, a novel approach to detect incipient slip based on the contact area between a transparent silicone medium and different objects using a neuromorphic event-based vision sensor (DAVIS) is proposed. Event-based algorithms are developed to detect incipient slip, slip, stress distribution and object vibration. Thirty-seven experiments were performed on five objects with different sizes, shapes, materials and weights to compare precision and response time of the proposed approach. The proposed approach is validated by using a high speed constitutional camera (1000 FPS). The results indicate that the sensor can detect incipient slippage with an average of 44.1 ms latency in unstructured environment for various objects. It is worth mentioning that the experiments were conducted in an uncontrolled experimental environment, therefore adding high noise levels that affected results significantly. However, eleven of the experiments had a detection latency below 10 ms which shows the capability of this method. The results are very promising and show a high potential of the sensor being used for manipulation applications especially in dynamic environments.

## 1. Introduction

Human dexterous abilities can optimally hold various objects without knowledge of shape, weight, and friction coefficient. Skin receptors in the human fingers acquire information of object slippage to apply minimum force during precision gripping [1,2]. Capability of the human touch sense inspires researchers to create artificial skins and various tactile sensors to enhance robots proficiency in human–robot interactions [3], manipulation tasks [4], and surgical applications [5].

Robotic grasping techniques are divided into two main categories, power grasp and precision grasp, whereas the former focuses on stability and the latter requires high sensitivity and precision [6]. Simple tasks for taking and placing specific tools in structured environments can be performed through power grasps. However, delicate manipulation for surgical applications in unstructured environments is required; therefore, the precision of the grasp method is essential in order to minimize risk of grasping failure. Moreover, extra applied force could result in object deformation or destruction; therefore, grasping nonrigid or breakable objects could require a more complex grasping model. Bicchi and Kumar [7] reviewed the literature on different power grasping methods and contact models, and indicated significant progress in structured grasping applications. Recent grasping techniques have simplified the force regulations by using tactile and position sensors considering unstructured environments with high uncertainty [8,9,10]. Detection of main parameters such as angular force, local strain and prediction of incipient slip based on the contact area facilitates grasping applications to perform adaptively regardless of object characteristics. To achieve acceptable and stable precision grasp, it is necessary to acquire contact area properties with low latency to feedback force into controllers and plan best grasping trajectories.

Tactile sensors are defined as devices to recognize contact status and exploit contact events. Various types of tactile sensors have been developed in the past, each of which has beneficial and negative impacts regarding the sensitivity, size, spatial resolution, and flexibility [11,12,13]. Piezoresistive tactile sensors consist of a conductive or semi-conductive elastomer that deforms under pressure. This deformation results in a change of material resistance that can be measured to identify touch and estimate the applied force [14,15]. Furthermore, Teshigawara et al. [16] proposed an application of Piezoresistive sensors to detect initial slip considering high frequency components of the output signal.

Capacitive tactile sensors are another well known category that considers variation of capacitance by changing load forces. These sensors comprise parallel conductive plates with dielectric in between, which can measure applied force on the plates by decreasing distance between plates, and, therefore, increasing the capacitance [17]. Similarly, Muhammad et al. [18] developed a capacitive tactile sensor array to recognize the texture pattern of the contact area.

Piezoelectrics are types of materials such as quartz, ceramics, and PolyVinyliDene Fluoride (PVDF) that generate electric flow under heat or deformation. This feature of piezoelectric materials has led researchers to compose a new generation of tactile sensors by placing a piezoelectric layer between two electrodes to measure generated voltage caused by the force load [19]. Goeger et al. [20] illustrated capability of PVDF tactile sensors to detect slip using short-time Fourier transform and k-nearest neighbour classifiers to identify slip states. They reconstructed images (8 × 128) pixels from PVDF output signal in order to use Eigenfaces algorithm to classify different states. Recently, Yu et al. [21] have developed a flexible piezoelectric tactile sensor array to measure force dynamically in three axes.

Moreover, some applications have measured the force vector through changes of magnetic properties. Takenawa [22] proposed a magnetic tactile sensor based on a two-dimensional array of inductors that can measure force vectors in three dimensions for detection of slippage. Furthermore, Goka et al. [23] added four Giant Magneto Resistance (GMR) chips to characterise load force and detect slip based on GMR’S output voltage. A more recent and interesting approach is presented in [24], using integrated magnetic tactile sensors with Hall-effect sensors to measure force vectors with high sensitivity and low hysteresis. Other methods such as Resistance Temperature Detectors (RTD) [25], acoustic [26], photoelastic, and friction-based [27] sensors were proposed to detect slippage and extract information of the object contact area [28].

Advancements in optical and digital image processing over the past decades have changed automation and industrial robotics applications drastically. Optical tactile sensors were introduced a long time ago to measure shear components of force for dexterous robotics applications [29]. Very early optical tactile sensors consisted of a transparent medium like silicate glass or rubber, LED light sources and a Charged-Coupled Device (CCD) camera [30,31]. They consider a source of light to emit beams on a rubber surface that has a direct contact with an object. Consequently, the camera records back-scattered beams and determines force vectors by comparing incidences and reflected angles. This method has been expanded over recent years to achieve higher sensitivity and optimize the cost of the sensors by using photo transistors and LEDs [32,33]. Kamiyama et al. [34] suggested an empirical method using a transparent elastic fingertip with spherical marks to track force angles and detect partial and incipient slippage. This approach has become more popular and similar sensors have been developed in [35,36,37,38,39].

Recent vision-based techniques acquire further information such as slip direction, surface texture, and object deformation. For instance, in [40], a vision-based slip sensor was developed that can identify object properties and slippage for deformable objects. They applied a Speeded Up Robust Features (SURF) algorithm to extract features of the contact area and match the feature points for both homogeneous and textured materials. Although camera-based sensors have made a significant progress in tactile sensing, improving sensor sensitivity, decreasing system latency and power consumption are necessary to meet industrial requirements.

Frame-based cameras have a considerable blind time between frames that limit the sensor latency and sensitivity. Even though high speed cameras can capture frames within less than one millisecond, they are less practical due to capturing redundant pixels, being expensive and having a high power consumption. Furthermore, capturing frames requires appropriate light conditions that limit the sensors performance in dark environments.

On the other hand, state-of-the-art neuromorphic cameras use retina silicone sensors to capture log-intensity brightness changes (events) in the scene rather than frames. Dynamic Vision Sensor (DVS) is an asynchronous event-based camera with high temporal contrast sensitivity to capture events efficiently in microseconds [41,42]. For instance, Ref. [43] developed a method to track object shape for force feedback estimation. Bio-inspired DVS is able to capture events in low light conditions while frame-based cameras could not obtain images in dark scenes. In this paper, we propose a novel event-driven incipient slip detection method using a Dynamic Active-pixel Vision Sensor (DAVIS) that can capture both events and log intensity frames. This developed sensor uses events to detect incipient slip, slip and vibration based on observing the object contact area with high sensitivity, low latency, and low power consumption in comparison to other vision-based methods.

## 2. Sensor Prototype

The functionality of the suggested sensor is based on monitoring the variation and displacement of the contact area between an object and a custom designed robot gripper. In [44], different stages of slip have been observed when moving a finger over a transparent piece of glass whilst measuring the changes in the contact area with a high speed camera (200 FPS). As a result, they demonstrated that the slip can be identified by monitoring the changes in the contact area, whereas a reduction of the contact area was observed prior to slip.

**Figure 1:**
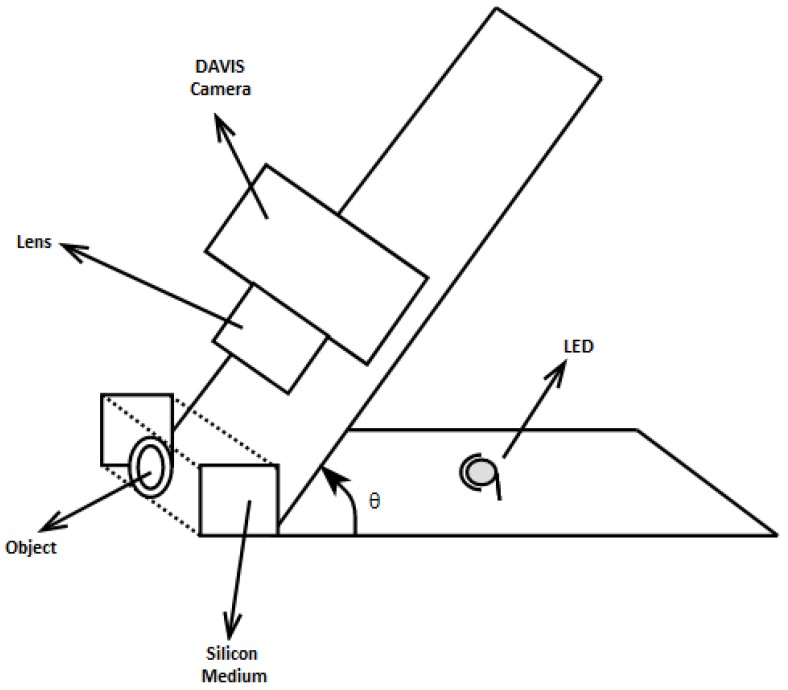
Sensor diagram.

In this paper, a method based on the same principle but adjusted to operate with events is proposed for identifying incipient slip according to relevant changes in the contact area using an event-based camera. In order to detect incipient slip with high sensitivity, it is necessary to eliminate noise since changes of local contact area can be slight at the start of incipient slip. For this purpose, the intensity trigger threshold of the sensor has been increased to reduce noise levels, thus acting as a high-pass filter. Figure 1 represents a diagram of the proposed sensor that includes a DAVIS camera (iniLabs, Zurich, Switzerland), silicone medium, source of light and mounting platform.

### 2.1. Silicone Medium Specifications

**Figure 2:**
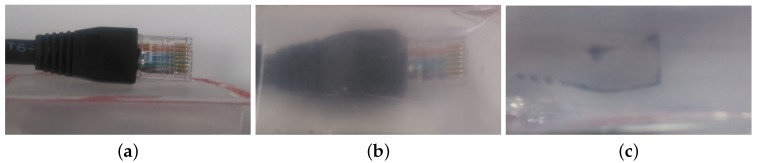
(**a**) contact of Ethernet cable with the silicone medium from the top; (**b**) direct observation through the silicone (0 degrees); (**c**) view of contact area considering 54 degrees to eliminate background noise.

A transparent elastic medium has been used to provide a flexibility to the sensor in order to identify concavity of the contact surface. The transparent silicone has been molded into a rectangular prism shape and placed between the object and camera. To extract the contact area of an object with the silicone medium surface and eliminate background noise, the DAVIS sensor has been deployed with θ angle to the vertical axis of the contact surface as shown in Figure 1. The angular view eliminates background noise due to light refraction. Figure 2 illustrates three different views of the contact between silicone medium and an object, whereas Figure 2a is the top view of an Ethernet cable in contact with the silicone surface, Figure 2b illustrates direct observation through the silicone medium, and Figure 2c shows the output when the camera has an angular view. As a result, Figure 2c provides clear observation of contact area whilst filtering out the background scene due to light refraction. This approach isolates the contact area from background and substantially improves the precision and reduces the computational time. A light source has been placed behind the silicone medium to reduce the impact of external illumination and enhance the visibility of the contact area. Furthermore, the silicone material is diffusive that also eliminates background noise.

### 2.2. Dynamic Active-Pixel Vision Sensor (DAVIS)

The DAVIS camera is a state-of-the-art hybrid camera that consists of both dynamic and active-pixel sensors that can record events and intensity frames. In this paper, we consider an event-driven solution instead of intensity frames to decrease sensor latency and optimize overall power consumption. Frame-based cameras use consistent power to capture frames while there are no changes in the scene. However, the event-based channel of the camera has a high temporal resolution of 12 μs for mean of 20 pixels and a low power consumption between 5–14 mW. For instance, a 4 mW power consumption is required when a hundred thousand events are triggered per second. This figure can be reduced to 2.6 mW when the scene has a thousand triggered events per second [41,42]. Furthermore, typical frame-based cameras consume more than 1 W to capture the scene. In [45], a CCD camera was used to detect object slip which typically has higher power-consumption than Active-Pixel Sensors (APS). As all the camera-based approaches in the literature used frame-based cameras to study the contact area, our proposed method consumes less power than other vision-based methods.

As mentioned earlier, the DVS camera fires events based on log-intensity brightness changes. The changes are continuously monitored and only fire ON/OFF events when the changes exceed ± trigger threshold. Timestamps of these asynchronous events are precisely measured with resolution up to 1 μs; however, timestamps increase to sub-ms for practical application. The DVS output includes precise time measurement (time-stamps), pixel positions (190 × 180) and polarity for each pixel. Moreover, a C-mount lens is used with the DAVIS camera with a focal length of 4.5 mm and iris range of F1.4. The size of each pixel is corresponding to approximately 0.126 × 0.126 mm in this setup considering a distance of 1.85 cm from the lens to the silicone medium surface. It is worth mentioning that changing the camera distance, lens properties, angle of camera, and silicone medium material can affect sensor resolution significantly.

Interestingly, in the DAVIS camera, DVS and Active-Pixel Sensors (APS) share the same photo-diode, whereas DAVIS has an added concurrent gray-scale frames output for each pixel. As a definition when the pixel value changes from its previous intensity to a higher value or lower value, it is referred as positive-polarity events or negative-polarity events, respectively. Polarity changes are important in event-based cameras as they can provide additional information of the scene. For instance, they can be used for understanding the movement direction of an object in an environment. Due to a high sampling rate of pixels, the sensor can obtain events continuously while frame-based cameras have a considerable blind time between frames.

In the proposed approach, the events polarity is used to identify pressure and slippage in two different stages that are described later in Section 3. This paper presents an event-driven incipient slip sensor to detect incipient slip, slip and vibration in low latency.

## 3. Image Processing

An image processing algorithm has been developed in this paper to reconstruct the contact area, estimate stress distribution and detect slip and vibration. As discussed in Section 2.2, the event-based camera captures events with positive and negative polarities that can be processed to estimate input force variation and shape of the contact area.

A robust algorithm should be able to filter various types of noise and generate robust results regardless of an object’s specifications. We performed initial experiments using different objects to analyse events precisely in grasping and releasing phases. Observations indicate that identifying incipient slip requires a certain amount of events to distinguish noise from events of interest. Each event represents one pixel in the scene; therefore, it is necessary to have several events in order to obtain meaningful features. Hence, we consider a fixed window of 10 ms to reconstruct a frame that includes all events triggered in that time period. All processes are performed on the reconstructed frames, which are denoted as event-based frames in this paper. Although the experimental environment can be controlled to reduce noise and improve sensitivity of the sensor, an ideal sensor should be robust to perform in unstructured environments.

The developed algorithm consists of two main parts: at first, reconstruct contact area and measure stress distribution. Secondly, identify vibration and slip. The following pseudo-code illustrates procedures of the proposed algorithm.

(1) Contact Area Reconstruction and Stress Distribution Map
**while**(startTime<startTime+10) **do**  // Create frames from events within 10ms**begin**posFrame ← **Read** positive eventsnegFrame ← **Read** negative eventsposFrame ← Morphology (posFrame)negFrame ← Morphology (negFrame)posFrameArea ← AreaFilter(posFrame)negFrameArea ← AreaFilter(negFrame)  **if**(negFrameArea>0) **then**  **begin**    contactArea ← negFrameArea    stressMap ← previousNegFrameArea + negFrameArea  **end****end**stressConcentrationPoint ← maxPoint(stressMap))

(2) Slip and Vibration Detection
**if**(posFrameArea>0) //Incident is detected**begin**  **if**(posFrameArea>= negFrameArea) then incident ← Slip  **else** incident ← Vibration**end**

### 3.1. Contact Area Reconstruction and Stress Distribution Map

**Figure 3:**
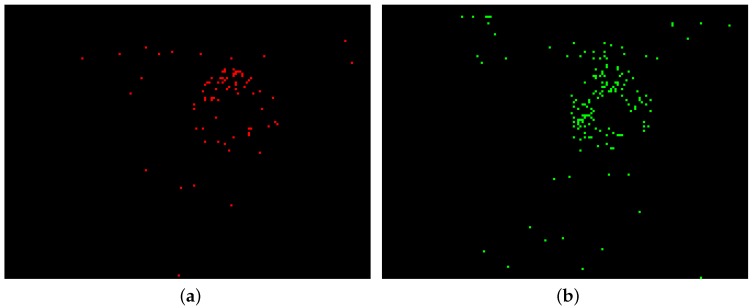
(**a**) negative-polarity events during the grasping phase; (**b**) positive-polarity events during the releasing phase.

In order to measure input force qualitatively in real time, it is required to analyse contact area characteristics during grasping phase. The increase of input force results in expanding the contact area as well as darkening this region. Therefore, the changes lead to triggering negative-polarity events. In contrast, during the releasing phase, a loss of contact area results in brightening these regions; therefore, it leads to trigger positive-polarity events. Figure 3a shows a 10 ms reconstructed frame from the negative-polarity events in the process of grasping an object and Figure 3b shows the positive-polarity events in the releasing phase.

**Figure 4:**
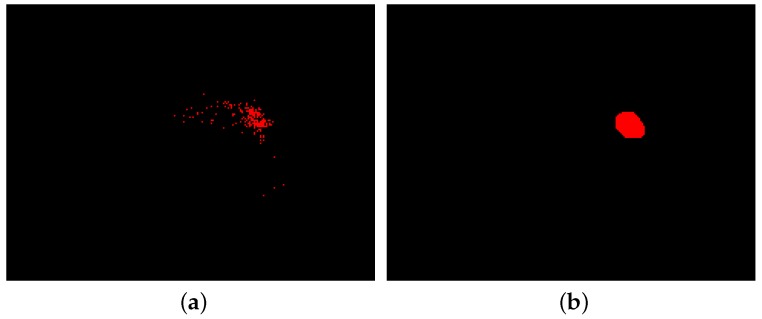
(**a**) negative-polarity events before filtering the noise; (**b**) detected region after filtering the noise.

Several factors affect the noise level in the images such as background noise, light variations, and silicone displacement during both grasping and releasing phases. As shown in Figure 3, a number of events have been triggered outside the contact area, which are considered as noise events. Since silicone medium has a diffusive surface, most of the background noise is eliminated physically and fewer noise events are triggered, which are widely scattered around the frame. In contrast, contact area events occur in specific regions with gregarious behavior. First, morphological operations based on the application of erosion and dilation masks are applied to remove any spurious pixels. Then, an area size filter is applied to identify and segment the largest connected areas above the minimum size. Morphological opening is accomplished by applying an erosion mask followed by a dilation while closing is performing by applying a dilation and then an erosion mask. The size of masks in the morphological operations can affect resolution of the sensor and, in the current algorithm, the resolution is 8 pixels, which is equivalent to 0.13 mm^2^. Figure 4 demonstrates the reconstructed frame before and after the noise filtering.

**Figure 5:**
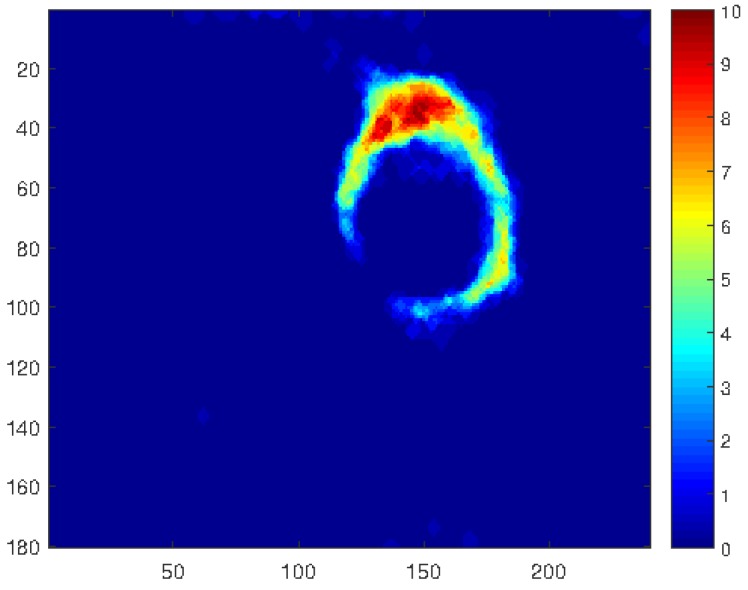
Reconstructed contact area with stress distribution map.

After filtering the noise, detected regions are considered to reconstruct the contact area. Furthermore, detected regions are accumulated into one frame in order to construct a stress distribution map. It is worth mentioning that high values in the stress distribution map indicate the high stress concentration points that can be investigated to analyse force and slip directions. Figure 5 demonstrates the qualitative reconstructed contact area with a stress distribution map scaling between 0 for the lowest and 10 for the highest stress points.

### 3.2. Slip and Vibration

In this research, incipient slip is considered as the earliest slight loss of the object contact area with the medium silicone and moving into the slip period, where the object contact area decreases continuously until it loses the whole contact to the silicone surface. Detection of incipient slip facilitates the system to prevent slip by modifying the gripping force. In addition, it is important to identify vibration for the following two reasons: (1) prevent a false-positive situation where vibration is detected as slip; (2) use detected vibration to correct trajectory plans and force feedback. In this paper, the vibration is referring to the specific object vibrations where it leads to change in the contact area. There are a lot of other parameters such as motor, arm, and camera vibrations that can affect the results which are out of the scope of this study.

**Figure 6:**
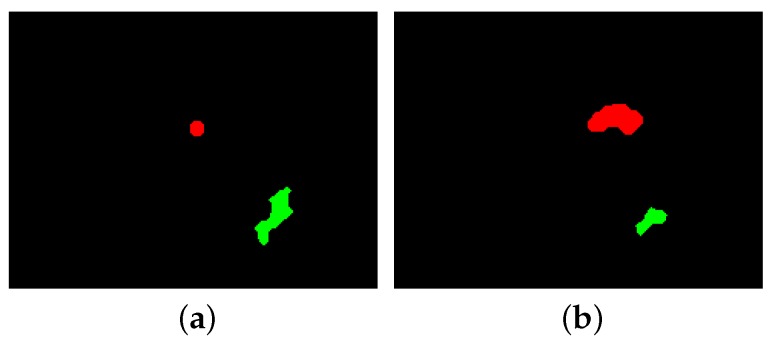
Green sections demonstrate positive-polarity areas and red regions represent negative-polarity areas. (**a**) incident is classified as slip; (**b**) incident is classified as vibration.

A reduction in contact area indicates slip occurring, which can be observed as positive-polarity events due to an increase in intensity. Size of detected area in positive-polarity events can evaluate the loss of contact area in each frame. This phenomenon is referred as ‘incident’ in this paper, where incident may be either slip or vibration. In vibration, both positive-polarity and negative-polarity events are observed. Clearly, when a negative-polarity area increases substantially, minor reduction of the contact area on the other regions could not result in object slip or dropping. Hence, if the negative polarity area is greater than the positive polarity area, the incident is classified as vibration; otherwise, it is classified as slip. For instance, in Figure 6b, the object has lost contact area from the bottom section shown in green while gaining more contact due to pressure on the top region shown in red. This means that the object has vibrated and the contact area has increased. In Figure 6a, the positive-polarity area is greater than the negative-polarity area, and, therefore, the incident is classified as slip.

**Figure 7:**
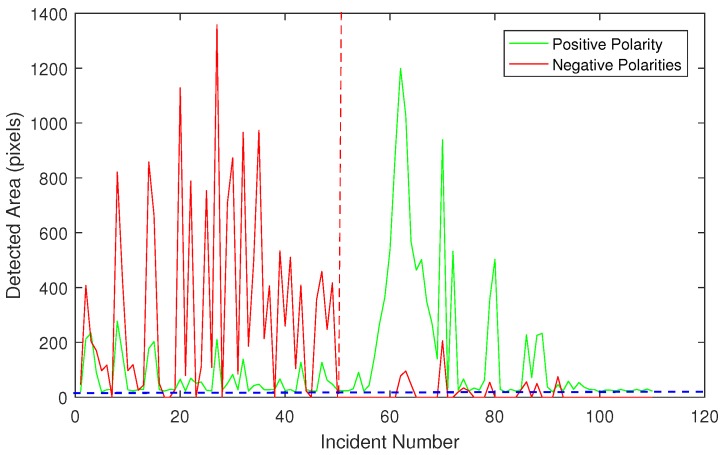
The red line shows negative-polarity and green illustrates positive-polarity changes, *x*-axis illustrates the incident number and *y*-axis shows the size of detected area.

In the proposed method, whenever positive-polarity areas are detected, further investigation is required to classify the incident. Figure 7 shows both positive and negative polarity areas for all detected incidents. As it can be observed, the first 50 incidents have been classified as vibration due to the negative-polarity area being greater than positive-polarity area. In contrast, after incident number 50, slip starts to occur, whereas the positive-polarity areas are greater than negative-polarity areas. Moreover, positive-polarity areas reach a peak at incident 65, which indicates a substantial loss of contact area at this point. Due to noise thresholding, small changes in the positive-polarity areas have been filtered, which is demonstrated by a dotted horizontal blue line in Figure 7.

## 4. Validation and Results

In this section, the experimental setup, stress distribution map, validation method using a high speed camera, and evaluation of slip sensor are presented and discussed in detail. Afterwards, the results are analysed in the discussion section.

### 4.1. Experimental Setup

**Figure 8:**
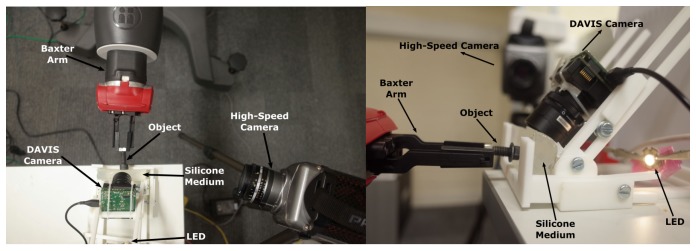
Top-down (**left**) and sideways (**right**) views of the experiment setup.

For validation purposes, thirty-seven experiments were performed using a Baxter robot (Rethink Robotics, Boston, MA, USA). In the experiment scenario, a force was applied to an object perpendicular to the surface of silicone by the robot arm, and then kept constant for a few seconds. Afterwards, the force from the robot arm was gradually reduced to release the object, whereas slip was observed. During this phase, object can be vibrated, which results in change of the object contact area. As the sensor considers only the contact area to detect slip, it is necessary to distinguish vibration and slippage incidents. As shown in the sensor diagram (Figure 1), the sensor includes an event-based camera, LED, silicone medium and adjustable camera frame. Figure 8 demonstrates experimental layout for the sensor components, high speed camera, Baxter robot arm, and the test object.

The adjustable sensor frame is a 3D printed platform that has been designed to mount the event-based camera and silicone medium. The platform enables linear and angular movement of the camera, in order to find the best angle to view the contact area and filter the background noise. The LED light increases the contrast on the surface of the contact area, which reduces the effect of background and environmental noise. A high speed camera is used to monitor the changes in the contact area independent of the event-based camera for validation purposes.

**Figure 9:**
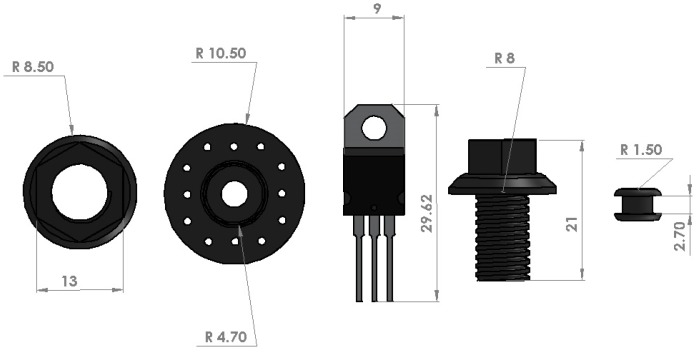
Objects used for the experiments including sizes in millimeters from left: nut, plastic servo head, IC, bolt, rubber grommet.

Five objects with dark colors were considered to perform the experiments. A variety of objects with different hardnesses, shapes and sizes have been chosen for the experiments to examine the viability of the proposed method. Figure 9 shows the shape and size for the used objects including metal nut, plastic servo head, Integrated Circuit (IC) made of aluminum and plastic, metal bolt, and rubber grommet.

### 4.2. Stress Distribution Map

**Figure 10:**
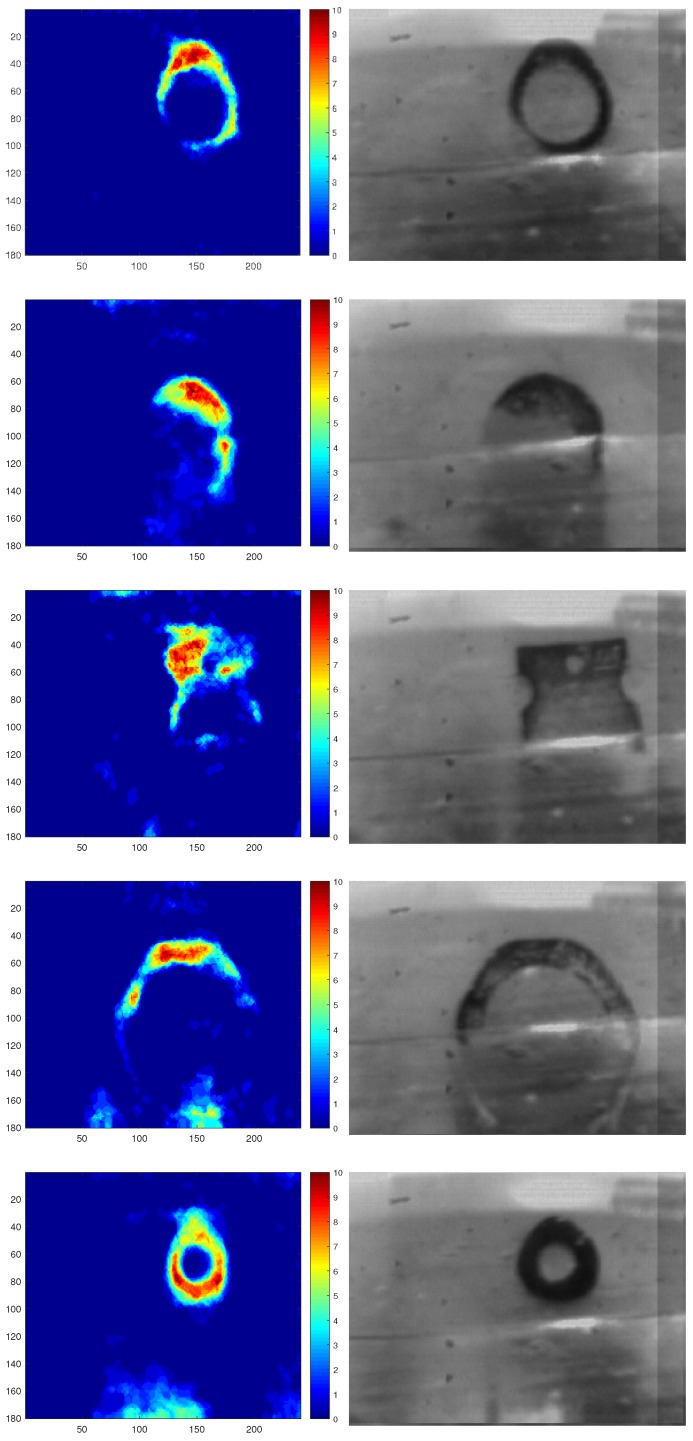
The first column shows the reconstructed contact area based on events. The second column illustrates the actual contact area from the concurrent frame output channel of the DAVIS camera.

As discussed in Section 3.1, a stress distribution map is reconstructed based on negative-polarity events. The stress concentration point is defined as the maximum value of pressure within the contact area. The left column in Figure 10 demonstrates reconstructed stress distribution maps for the five objects, which are normalized between 0 to 10. Moreover, the second column in the figure illustrates the actual contact area that is captured by the concurrent frame output channel of the DAVIS camera.

The results show that the contact area is reconstructed satisfactorily. In the proposed method, further information is extracted such as stress distribution throughout the contact area. The stress distribution map provides an understanding of crucial stress points, which can be used to estimate the consequent force vector. These features provide further information of incidents, and, therefore, the system can modify the force and trajectories more efficiently.

### 4.3. High Speed Camera and Synchronization

The performance of the proposed slip sensor using an event-based camera (DAVIS) can be affected by light noises in the contact area and background. Therefore, we consider using a conventional high speed camera as a validation method to study the contact area visually.

**Figure 11:**
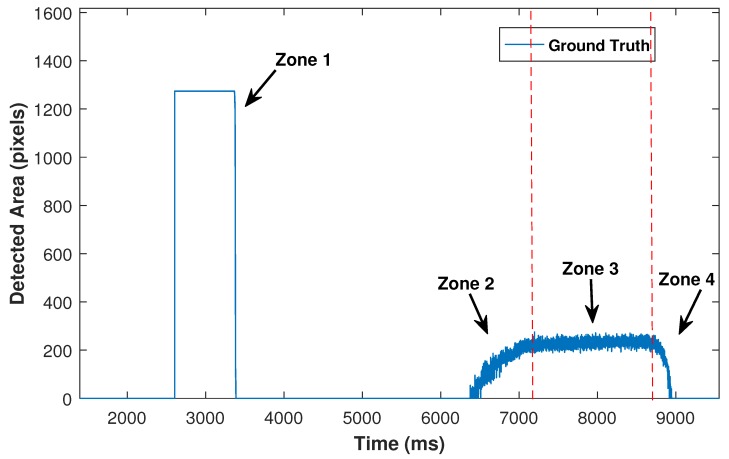
Detected area by high speed camera. (1) synchronisation phase; (2) increasing force to grasp the object; (3) holding the object; (4) releasing phase.

A high speed camera (1000 frames per second) is used to validate the proposed method, viewing the contact area from a different angle compared to the event-based camera. Ground truth contact areas are estimated by applying image thresholding on the high speed camera frames. Figure 11 demonstrates the detected area by the high speed camera over time. Zone 1 represents the synchronisation phase where an LED blinks to allow synchronisation between the high speed and the event based cameras. Zone 2 illustrates the increase of contact area for the grasping phase, which is caused by increasing input force from the robot. Zone 3 is where the applied force is constant, and zone 4 displays the releasing phase as the contact area is decreased by the reduction of input force. The start of zone 4 is known as incipient slip, which is followed by slip incidents.

In the releasing phase, the slip starting point (incipient slip) and final point where no contact area is detected are used for validation of slip results. To identify the exact time of incipient slip, the changes in the contact area are monitored in the zone 3 (with constant applied force), where the minimum detected area size is found. In the releasing phase, whenever the contact area decreases to less than the minimum detected area in zone 3, incipient slip has started. Clearly, slip events continue until the detected area size becomes zero.

### 4.4. Evaluation of the Slip Sensor

To evaluate the sensor performance, the results obtained from the event-based camera have been benchmarked against a high speed camera. As discussed in Section 4.3, the results from the event-based camera are synchronized with the high speed camera. The validation method is based on the precision and recall (i.e., sensitivity) method whereas the number of True-Positive (TP), False-Positive (FP) and False-Negative (FN) situations have been considered. In this approach, True-Negatives (TNs) are ignored, as their huge number skew the results.

**Figure 12:**
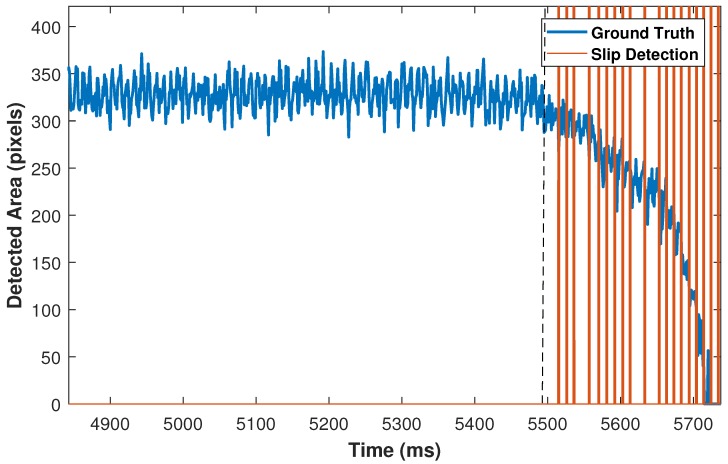
Ground truth: blue, start of incipient slip: dotted line, TP incidents: red lines, FN incidents: abnormal gap after dotted line.

To measure the number of TP and FP situations, all frames from the start of incipient slip are investigated until the object no longer has any contact with the silicone medium. For validation purposes, the time is split into 20 ms frames. For each frame, a TP condition occurs when slip happens and the sensor detects it correctly. However, if, in such a circumstance the sensor is not able to detect the slip, the incident is considered as FN. If the sensor detects slip incorrectly while no actual slip exists, this incident counts as an FP situation. Figure 12 illustrates ground truth results that have been obtained from the high speed camera in blue. The high speed camera results have been synchronized with the proposed sensor, whereas red lines indicate TP detections, and the abnormal gap between slip incidents during the releasing phase represents FN incidents. Furthermore, the time between the dotted line (start of slip) and the first slip incident is defined as sensor latency.

**Figure 13:**
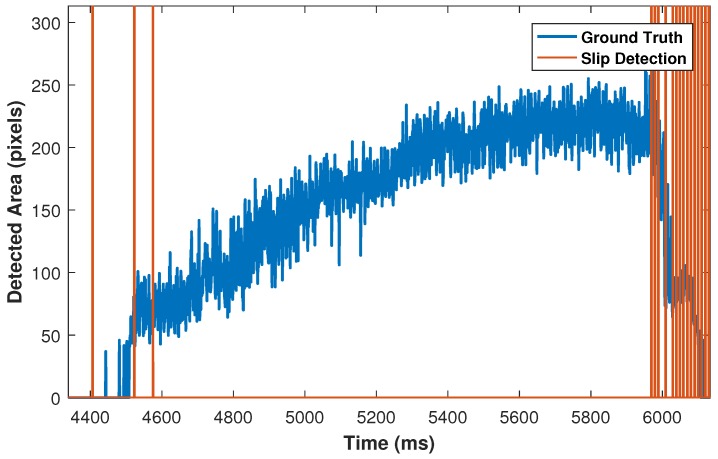
First three slip incidents represent FP conditions.

Similarly, TP and FN slip incidents can be observed in right side of Figure 13 during the releasing phase. However, three slip incidents have been detected during the grasping phase that represent FP conditions.

Furthermore, TP, FP and FN can be taken into account to calculate sensitivity (1), precision (2) and F1 score (3) by the following formulas:

(1) Sensitivity=TP/(TP+FN),

(2) Precision=TP/(TP+FP),

(3) F1=2TP/(2TP+FP+FN).

In order to measure the sensor latency, the time between the start of incipient slip and the first TP detection is measured.

Average results obtained from thirty-seven experiments are summarized in Table 1, including Precision, Sensitivity, F1 Score, and Latency. F1 score can indicate the relation of average precision and sensitivity for the achieved results. Moreover, average standard deviation for latency results of each object is denoted as σ.

## 5. Discussion

The objects can be classified into three categories based on material hardness, whereas nut and bolt are considered hard metals, the IC and Servo head as plastics, and the washer as a flexible rubber. It can be observed from Table 1 that the two objects with the highest sensitivity and lowest latency are the Nut and Bolt. Therefore, the metal objects have shown a better performance due to the friction and physical adhesion. It is worth reminding readers that parameters such as the size of contact area, object shape, direction of input force, and level of contrast between silicone medium and the object can also affect the results slightly.

On the other hand, the worst results were achieved from the plastic Servo head in all experiments. The small inner ring of the servo head is in contact with the silicone medium, and, therefore, it produces a smaller contact area. This can be one of the main reasons for the high latency. The rubber grommet was the smallest object, thus it was expected to achieve unfavorable results. However, the rubber grommet is a soft object and it expands its contact area in grasping phase. Hence, the results for the rubber grommet have achieved higher sensitivity and lower latency than excepted.

A lower detection latency provides more time for the robot gripper to respond to slippage. Therefore, one of the main factors in slip prevention is to detect latency of incipient slip. In [37,40], cameras sampling rate are 82 FPS (12.2 ms) and 30 FPS (33.3 ms), respectively. However, they did not provide the latency of sensors detection. Moreover, a flexible capacitive polymer tactile sensor was proposed in [46] with sampling rate of 6 FPS (166.6 ms) and sensor response time of 160 ms. In this paper, the proposed sensor reconstructs frames from the events with sampling rate of 100 FPS (10 ms) and average of 44.1 ms latency, which shows enhancement in sampling rate and latency compared to the methods mentioned above. To the best of the authors’ knowledge, this research study proposes the first approach for slip detection using an event-driven camera. This method is based on monitoring the changes in the contact area. Use of events instead of frames can enhance power consumption and latency in vision-based slip sensors criteria. Notably, eleven of the experiments had a detection latency below 10 ms, which shows the capability of the sensor for real-time applications and future improvements. It is worth mentioning that the achieved latency should be improved since other tactile sensors such as illustrated in [47], and they demonstrated low latency of 1–3 ms with high resolution and a 100 FPS sampling rate.

According to Table 1, the standard deviation of detection latency for each object has widely varied over experiments. The reason is that experiments were not performed under a strictly controlled environment and have been influenced by several factors such as vibration, force direction, and robot arm acceleration. Moreover, the experiment’s scenario is designed to test the novel approach for incipient slip detection, and it requires further experiments to evaluate stability of the system and performance of the sensor precisely.

In [37], a vision-based slip margin feedback was proposed for a elastic object with the displacement resolution between $10^{1}$ to $10^{3}$ pixel/mm. Moreover, in [48], a flexible 16 × 16 tactile sensor array was proposed for invasive surgery applications with a high spatial resolution of 1 mm and non-steady output. Furthermore, in [49], resolution of 0.1 mm is reported for localisation of the force. As mentioned in Section 2.2 and Section 3.1, each pixel corresponds to 0.126 × 0.126 mm and the sensor has equivalent resolution of 0.13 mm^2^. For different applications, the resolution of the system can be modified by changing camera distance, lens properties, silicone material and morphological operations.

**Figure 14:**
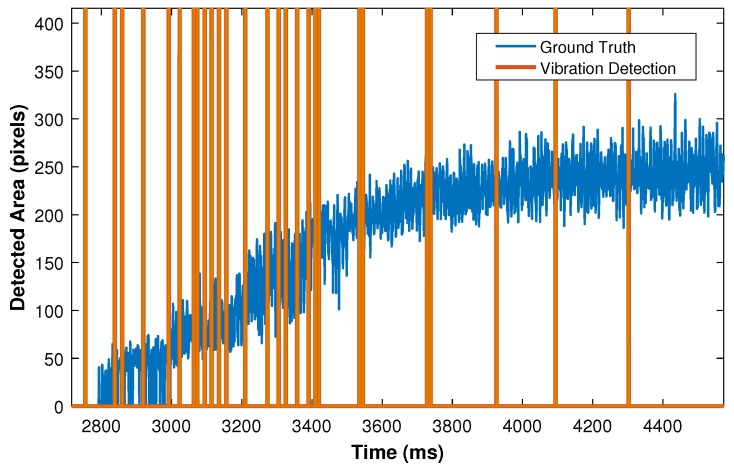
Detection of vibration incidents during grasping phase.

It is important to note that this research study considers parameters that influence only the object vibrations. Figure 14 illustrates vibration incidents detected by the developed approach in the grasping phase. As it can be seen, several vibration incidents have been detected in the beginning of grasping phase since this part involves severe vibrations. In contrast, when the system is moving towards the constant force phase, vibration is reduced in the gripper. As a result, the sensor has correctly detected fewer vibration incidents.

## 6. Conclusions

In this paper, a novel methodology to detect incipient slip using neuromorphic event-based vision sensor (DAVIS) is demonstrated. The results show that the proposed methodology is very efficient and able to detect the incipient slip using different objects’ materials and sizes with average of 44.1 ms latency and satisfactory resolution. Therefore, it can be applied for industrial applications where the robot manipulator has to handle a variety of objects without prior knowledge of their specifications. Challenges such as estimating force quantitatively and evaluating the proposed sensor by using force sensors can be studied in the future. Furthermore, designing new scenarios and performing more experiments are necessary to study vibration of the system and stability precisely.

## Figures and Tables

**Table 1:** Algorithm average results for precision, F1 score, Sensitivity, and Latency (δ = standard deviation).

Object	Precision	Sensitivity	F1 Score	Detection Latency (ms) and σ
Bolt	0.71	0.97	0.80	34.5 (σ = 49.0)
Servo Head	0.63	0.71	0.65	73.6 (σ = 56.1)
Rubber Grommet	0.66	0.87	0.73	41.2 (σ = 29.2)
Nut	0.65	0.90	0.73	32.0 (σ = 48.1)
IC	0.85	0.80	0.82	39.4 (σ = 45.2)
Average	0.70	0.85	0.75	44.1 (σ = 45.5)
